# Treatment of inhalation injury by nebulization of mesenchymal stem cell-derived exosomes

**DOI:** 10.1097/JS9.0000000000004700

**Published:** 2026-01-20

**Authors:** Zhongzhao Wang, Yu Jin, Chao Zhou, Xintong Du, Jian Wang, Hao Tang

**Affiliations:** a Department of Respiratory and Critical Care Medicine, Changzheng Hospital, Second Affiliated Hospital of Naval Medical University, Shanghai, China; b Department of Respiratory and Critical Care Medicine, Changhai Hospital, First Affiliated Hospital of Naval Medical University, Shanghai, China; c ANEXT Shanghai Biotechnology Co., Ltd, Shanghai, China

## Background

All authors confirm that no artificial intelligence tools were used in the preparation of this manuscript[1]. Inhalation injury is common in burn patients and significantly increases mortality in burn patients[2]. Nevertheless, the treatment of inhalation injury remains supports mainly including mechanical ventilation, anti-inflammatory agents, bronchodilators, mucolytic agents, and anticoagulants[3]. The mesenchymal stem cell-derived exosomes (MSC-Exos) used for treatment of acute lung injury has shown promise in preclinical trails[4]. MSC-Exos activate the nuclear factor erythroid 2-related factor 2 (Nrf2)/HO-1 pathway, inhibit the phosphorylation of nuclear factor-κB (NF-κB) and the nuclear translocation of p65, promote the phosphorylation of Akt and the nuclear translocation of Nrf2, and enhance Akt/Nrf2/HO-1 signaling, thereby reducing oxidative stress and ferroptosis in acute lung injury[5].MSC-Exos is capable of treating respiratory diseases including COVID-19, acute respiratory distress syndrome, and pulmonary infection in preclinical experiment[6]. Meanwhile, clinical trials have preliminarily proved the safety of nebulization of MSC-Exos[7]. However, to our knowledge, there are no reports on the treatment of inhalation injury by nebulization of MSC-Exos.

## Case report

A 77-year-old Chinese woman with multiple myeloma was diagnosed with severe critical pneumonia. After more than a month of tracheal intubation, she was found upper tracheal stenosis and underwent attempted tracheostomy placement. The procedure was complicated by a spark generated during the electrocoagulation process leading to smoke generation and iatrogenic inhalational lung injury to the patient (Fig. 2A, F, Supplemental Digital Content Video 1, available at: http://links.lww.com/JS9/G627).

**Figure 1.**
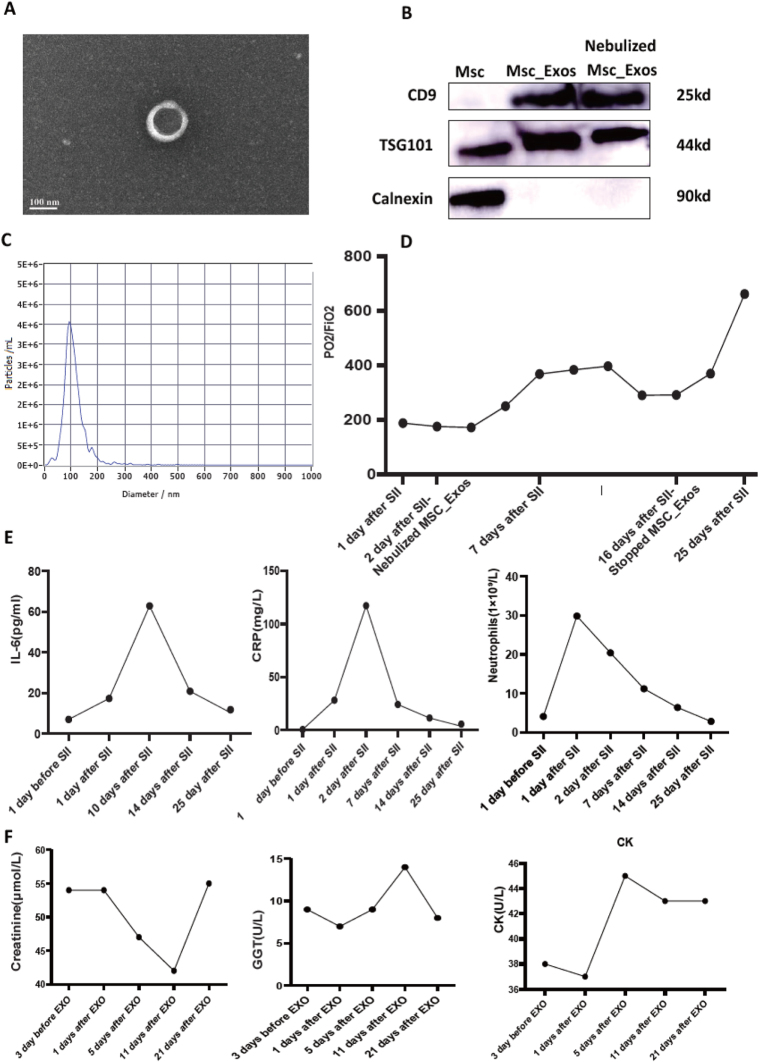
(A) Transmission electron microscopy (TEM) images show intact mesenchymal stem cell-derived exosomes (MSC-Exos). (B) Western Blot (WB) results show that MSC-Exos highly express CD9 and TSG101, indicating high purity and definite identity. The absence of Calnexin expression suggests no endoplasmic reticulum contamination. Nebulization does not affect the intactness of MSC-Exos. (C) Particle size analysis indicates that the diameter distribution of nanoparticles is 100 to 200 nm, which is consistent with the size range of exosomes. (D) Results of arterial blood gas analysis show that the oxygenation index (PaO₂/FiO₂ ratio) of patients increases significantly after MSC-Exos administration. (E) Serum interleukin-6 (IL-6), C-reactive protein (CRP), and neutrophils number all decrease significantly after treatment, demonstrating that the inflammatory state of patients is significantly relieved after treatment. (F) Liver and kidney functions, as well as cardiac injury-related indicators of patients, are all within the normal range before and after treatment, confirming the safety of MSC-Exos. Abbreviations: SII, severe inhalation injury; MSC-Exos, mesenchymal stem cell-derived exosomes; IL-6, interleukin-6; CRP, C-reactive protein; GGT, gamma-glutamyl transferase; CR, creatinine.

**Figure 2.**
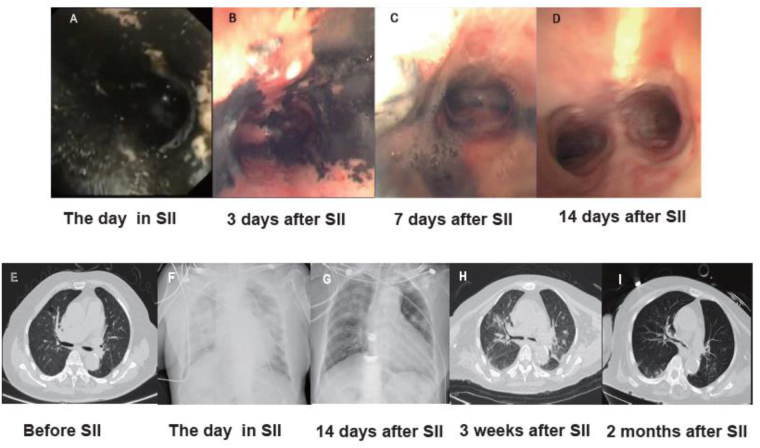
Bronchoscopic image of bronchial tube and pulmonary imaging. (A) Bronchoscopy revealed abundant black soot and serious injury of bronchial tube in the day of inhalation injury. (B) Bronchoscopy showed still seriously injured bronchial tube of patient after 3 days of inhalation injury. (C) Hyper mucus secretion was observed in bronchoscopy after 7 days of inhalation injury (D) Mucus had disappeared and wound surface of bronchial inhalation injury recovered basically including reduced mucosal edema and hemorrhage, after 10 days treatment of mesenchymal stem cell exosome nebulization. (E) Chest computed tomography showed the pulmonary condition of patient in 5 months before inhalation injury. (F) Chest X-ray revealed the severe inhalation injury in the day of inhalation injury. (G) Chest X-ray on the tenth days after inhalation injury revealed effective treatment of mesenchymal stem cell exosome nebulization. (H) Chest computed tomography showed recovery of lung in the day after 3 weeks of inhalation injury. (I) Chest computed tomography in 2 months’ follow-up revealed good recovery of pulmonary parenchyma and no severe pulmonary fibrosis. Abbreviation: SII, severe inhalation injury.

Her condition was critical showing oxygen saturation was 85% without ventilator and PO_2_/FiO_2_ ratio was 189(Fig. 1D). The patient was granted compassionate use of MSC-Exos by ethics committee. Nebulization of 5 mL of MSC-Exos (1 × 10^7^ particles/mL) (Fig. 1A, C) was given once a day for 2 weeks. At the same time, ventilator-assisted ventilation, anti-inflammatory, airway atomization with budesonide and acetylcysteine, and symptomatic treatment with antibiotics were provided. The patient’s condition gradually stabilized, in 3 days (Fig. 1D, Fig. 2B, Supplemental Digital Content Video 2, available at: http://links.lww.com/JS9/G628). Tracheoscopy was performed to clear the airway. On the seventh day, the patient’s oxygen saturation was maintained 94% without ventilator (Fig. 1D, E, Fig. 2C, Supplemental Digital Content Video 3, available at: http://links.lww.com/JS9/G629). Two weeks later, the nebulization of MSC-Exos was stopped, and tracheoscopy revealed significant resolution of mucosal inflammation (Fig. 2D, Supplemental Digital Content Video 4, available at: http://links.lww.com/JS9/G630). No significant adverse events were observed (Fig. 1F). Follow-up chest CT in 2 months revealed no severe pulmonary fibrosis (Fig. 2I).

## Discussion

We report the clinical course of a patient with severe inhalation injury who was successfully treated by nebulization of MSC-Exos. Previous research showed length of stay in the intensive care unit (ICU) for III to IV degree inhalation injury patients is about 12 days[8]. In another prospective study, the control inhalation injury group included 70.5% of patients with mild-to-moderate inhalation injuries. The duration of ventilator use within 28 days was as high as 17.5 days, and the mean time in the ICU was as long as 20.5 days[9]. However, in this severe patient, vital signs were basically stable just 3 days after treatment, and the patient met the criteria for discharge from the ICU in only 7 days. These findings suggest that MSC-Exos are safe and may have therapeutic potential.

Inhalation injury is really rare and high disease heterogeneity. The treatment was affected to varying degrees of body surface burns. Therefore, over the past few decades, there have been very few prospective studies on inhalation injuries. We used nebulization of MSC-Exos to treat inhalation injury for the first time and found that MSC-Exos were safe and might have therapeutic potential for inhalation injury which warranted further investigation in controlled clinical trials. Our case may provide reference for the treatment of inhalation injuries in the future.

## Data Availability

Data Sharing Statement Data available: No Sharing such data would enable the identification of the patient. We are obligated by our Institutional Review Board (IRB) protocol to maintain the confidentiality of our study patient.

## Research registration unique identifying number (UIN)

Not applied.
